# A multicenter, prospective cohort study on the anti-SARS-CoV-2 vaccination response in patients with multiple sclerosis in Germany

**DOI:** 10.3389/fneur.2025.1674742

**Published:** 2025-10-29

**Authors:** Achim Berthele, Clemens Gödel, Boris-Alexander Kallmann, Markus Kowarik, Klaus Lehmann-Horn, Martin Marziniak, Sebastian Rauer, Veit Rothhammer, Florian Then Bergh, Mathias Wahl, Brigitte Wildemann, Ksenija Schirduan

**Affiliations:** ^1^Department of Neurology, School of Medicine and Health, Technical University Munich, München, Germany; ^2^Department of Neurology, Leipzig University, Leipzig, Germany; ^3^MS Center Bamberg (mszb), Bamberg, Germany; ^4^Department of Neurology and Stroke, Hertie-Institute for Clinical Brain Research, University of Tübingen, Tübingen, Germany; ^5^Neuropraxis München Süd, Unterhaching, Germany; ^6^Department of Neurology, kbo-Isar-Amper-Klinikum München-Ost, Haar, Germany; ^7^Department of Neurology and Neuroscience, Faculty of Medicine, Medical Center University of Freiburg, University of Freiburg, Freiburg, Germany; ^8^Department of Neurology, University Hospital Erlangen, Erlangen, Germany; ^9^Department of Neurology, DKD Helios Klinik Wiesbaden, Wiesbaden, Germany; ^10^Department of Neurology, Molecular Neuroimmunology Group, University Hospital Heidelberg, Heidelberg, Germany; ^11^Biogen GmbH, München, Germany

**Keywords:** multiple sclerosis, SARS-CoV-2, vaccination, immune response, disease-modifying therapy

## Abstract

**Background:**

This epidemiologic cohort study documented clinical and serological data in MS patients over several vaccination cycles against severe respiratory syndrome coronavirus-2 (SARS-CoV-2) in a real-world setting.

**Methods:**

Adult patients with MS were included during a period of 26 months from July 2021 if SARS-CoV-2 vaccination was planned, or first dose was given, or vaccination was completed within the last 6 weeks, or vaccination was completed >6 weeks ago and a booster dose was planned within the next 90 days. Humoral immune response to authorized SARS-CoV-2 vaccines was investigated during each vaccination cycle at baseline and approximately 1 and 6 months after vaccination. Immune response was defined as an anti-SARS-CoV-2 spike protein IgG titer >100 BAU/ml above pre-vaccination level and, separately, by the presence of SARS-CoV-2 neutralizing antibodies (NAb) approximately 1 month after the last vaccination.

**Results:**

Of 159 patients enrolled, 140 (88.1%) were being treated with a DMT. Most patients (67.9%, *n* = 108) entered the study after complete initial SARS-CoV-2 vaccination (up to two doses) and before the 1st booster dose. Approximately 1 month after the 1st booster vaccination, response was seen in 68.1% of the patients (*n* = 79/116) based on anti-S1-IgG increase and in 72.1% (*n* = 88/122) based on NAb seropositivity. Persisting immune response approximately 6 months after vaccination was observed in 71.8% (*n* = 51/71) and in 93.7% (*n* = 74/79) of the responders, respectively. Adequate humoral immune response and persistence of response was less frequent in patients on anti-CD20 antibodies or sphingosine-1-phosphate receptor (S1PR) modulators compared to patients on other DMTs or DMT-untreated patients. Breakthrough infections with the SARS-CoV-2 virus were reported in 58 patients (36.5%). Seven patients (4.4%) experienced an MS relapse during the study period.

**Conclusions:**

With the exception of anti-CD20 antibodies and S1PR modulators, DMTs did not impair humoral response to any of the authorized SARS-CoV-2 vaccines. Persistence of humoral immune response was seen over a period of at least 3 months in the majority of initial responders but was decreased in the anti-CD20 antibodies/S1PR modulator subgroup.

**Clinical trial registration:**

This epidemiological study is registered in the German Clinical Trials Register (DRKS00025893).

## 1 Introduction

Since the outbreak of the coronavirus disease 2019 (COVID-19) pandemic in 2020, patients with multiple sclerosis (MS) and their physicians have faced additional challenges with regard to the increased risk of infection on the one hand and the need for effective treatment of MS on the other hand. Multiple sclerosis (MS) is an autoimmune-mediated inflammatory disease of the central nervous system (CNS) characterized by areas of demyelination and axonal degeneration (1–3). Although the exact causes of MS are still unclear, lesion formation is the result of infiltration of peripheral immune cells into the CNS, which triggers a series of events leading to activation of endothelial cells, recruitment of additional lymphocytes and monocytes, release of proinflammatory cytokines and subsequent demyelination (4). The cornerstone of MS treatment is therefore the suppression or modulation of the immune system. Various disease-modifying therapies (DMTs), which target the immune system via different modes of action, have been approved for the treatment of MS in Germany. These include first-generation DMTs such as injectable interferon beta and glatiramer acetate as well as highly effective second-generation therapies such as monoclonal antibodies (i.e., natalizumab, anti-CD20 B-cell depleting antibodies), sphingosine-1-phosphate receptor (S1PR) modulators, teriflunomide, cladribine, and fumarates. However, these second-generation DMTs in particular have been associated to various degrees with a higher risk of community-acquired and other types of infection (5–7).

As a general immune defense, vaccinations aim to generate long-term responses and thus valuable protection against pathogens. Reducing the severity of illness and preventing deaths through the protection of a vaccination was particularly important in the context of the COVID-19 pandemic, caused by the severe respiratory syndrome coronavirus-2 (SARS-CoV-2). Published data show differences in the immune response in MS patients, depending on the type of pathogen vaccinated against, the type of vaccination, or the type of immunotherapy given to the patient (8–11). To make a balanced decision between the most beneficial MS therapy for the individual patient and the expected response to an upcoming vaccination, including SARS-CoV-2 vaccination, information about the patient's immunization status is extremely valuable.

This epidemiological study was conducted to collect clinical and serological data before and after SARS-CoV-2 vaccination over several vaccination cycles of individual patients in a real-life setting, and to evaluate the possible effects of different DMTs on the patients' immunization status. This study aimed to provide a basis for counseling and treatment of MS patients in the pandemic situation and beyond.

## 2 Methods

### 2.1 Study design and ethics

**Figure 1:**
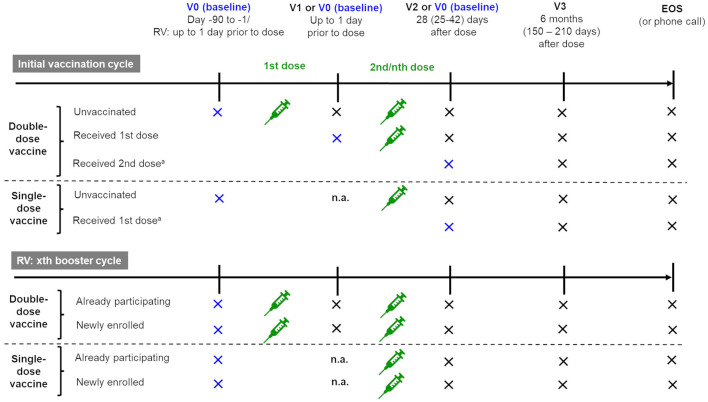
Visit schedule per vaccination cycle (initial and booster cycles) depending on the patient's vaccination status at enrollment. EOS, end of study; n.a., not applicable; RV, repeat vaccination; V, visit.

This prospective epidemiologic cohort study was performed at 10 neurological or MS centers in Germany between July 2021 and September 2023. Individual patients could be followed up over several vaccination cycles during the study period. The decision to be vaccinated against SARS-CoV-2 (initial or booster vaccination) was made by the patient independently of the study and, if necessary, in consultation with the treating physician. The concept of this observational study and its documentation procedure did not influence the routine treatment situation. The study visits were combined with the patient's routine clinical visits. Figure 1 illustrates the planned visit schedules for initial and booster SARS-CoV-2 vaccination cycles. The schedule depended on the SARS-CoV-2 vaccination status of the patient at enrollment and whether a single- or double-dose vaccine was used. Each cycle started with a baseline visit (V0) prior to the first SARS-CoV-2 vaccination of this cycle. V0 was followed by up to three post-dose follow-up visits (V1, V2, and V3) within a period of approximately 6 months. In the initial vaccination cycle, V0 could also be performed after the first or second dose.

All patients gave their written informed consent for their data to be documented and analyzed as part of the study. All data were collected in a pseudonymous way, whereby patients could only be identified by their respective participating physician via a unique identification code. The study was conducted in accordance with the ethical principles established in the Declaration of Helsinki and all applicable national regulatory requirements. The study was reviewed by a competent ethics committee and was registered with the Federal Institute for Vaccines and Biomedicines (Paul Ehrlich Institute) and the German Clinical Trials Register (DRKS).

### 2.2 Study population

Patients were recruited by participating MS centers as part of their daily clinical routine. Both patients who were currently being treated with a DMT for MS and those who were not being treated with a DMT were eligible for inclusion. Therapy decisions were made independently of the study and could change during the course of the study. To achieve balanced recruitment, patients were stratified according to their MS DMT treatment at the time of inclusion in the study. Inclusion criteria were: (a) male or female ≥18 years of age; (b) diagnosis of MS according to McDonald criteria (2018) (12); (c) ability to understand the purpose of the study and provide signed and dated study-specific informed consent; (d) SARS-CoV-2 vaccination planned within the next 90 days, or first of two doses received, or first vaccination cycle completed within the last 6 weeks, or vaccination completed >6 weeks ago and booster dose planned within the next 90 days; (e) in case of prior SARS-CoV-2 infection(s), patient has recovered from infection. Patients were excluded if regular follow-up was not possible for organizational or geographical reasons and/or in the event of unwillingness to be vaccinated against the SARS-CoV-2 virus.

### 2.3 Data collection

Data were collected during routine visits and/or from patients' medical records. The following data were documented at enrollment (baseline): age, sex, height, weight, comorbidities, smoking habits, MS onset date, MS disease characteristics, including most recent score on the Expanded Disability Rating Scale (EDSS) (13), occurrence of MS relapse episodes within the last 6 months, and high-dose corticosteroid (HDC) therapy for relapse with therapy dates. Other documented data were current and previous MS treatment within the last 12 months, recent laboratory data (differential blood count, serum immunoglobulin levels), general immunization status taken from the patients' vaccination certificates (vaccination dates were not documented), SARS-CoV-2 infection history, SARS-CoV-2 vaccination status, and information on previous SARS-CoV-2 vaccinations. At the repeated baseline visits of the following vaccination cycles, recent laboratory data, information on current MS treatment, MS disease characteristics, immunization status, and SARS-CoV-2 infection were collected. At the follow-up visits during each cycle, changes in MS medication, MS disease characteristics, recent differential blood count results, and information on SARS-CoV-2 infection, SARS-CoV-2 vaccination, and changes in the immunization status since the last visit were recorded.

### 2.4 Laboratory assessments

Blood samples (8 ml) for the following serological parameters were taken at baseline (V0) and all post-baseline visits (V1, V2, and V3) of a cycle: anti-SARS-CoV-2 spike protein (S1 domain) antibodies (IgA, IgG), anti-SARS-CoV-2 nucleocapsid protein (NCP) antibodies (IgG), SARS-CoV-2 S1-IgG neutralizing antibodies (NAb), and anti-influenza A virus antibodies. Blood samples were centrifuged and stored locally at below −18 °C until shipment on dry ice to the central laboratory (Unilabs AB, Copenhagen, Denmark). Quantitative detection of SARS-CoV-2-specific immunoglobulins (anti-S1-IgA, anti-S1-IgG, and anti-NCP-IgG) was performed by using the Anti-SARS-CoV-2 ELISA (IgA), Anti-SARS-CoV-2-QuantiVac ELSA (IgG), and the Anti-SARS-CoV-2 NCP ELISA (IgG) tests (Euroimmun Medizinische Labordiagnostika AG, Lübeck, Germany). The SARS-CoV-2 NeutraLISA (Euroimmun) neutralizing assay was used to test the neutralizing activity of the anti-S1-IgG. Anti-influenza A virus IgG antibodies were analyzed by using the Anti-influenza A virus ELISA (Euroimmun).

### 2.5 Data analysis and statistics

For the purpose of data analysis, the visits were derived for each cycle from the documented visit dates. To obtain the highest possible number of evaluable patients, visit windows were slightly extended compared to the original schedule as planned in the protocol and shown in Figure 1. The following time windows were used in the analysis: up to the same day as the 1st initial/nth booster vaccination (V0), up to the same day as the 2nd initial/2nd dose of nth booster vaccination (V1), 28 days (range: 15–56 days) after initial vaccination completed/nth booster vaccination (V2), 6 months (range: 136–274 days) after initial vaccination completed/nth booster vaccination (V3). Baseline was defined as a time point before (first) SARS-CoV-2 vaccination of a cycle. If V0 had not been performed before a booster vaccination or respective data were not available for this visit, V3 of the previous cycle was used as baseline in case the visit date was within the defined time window. Vice versa, if V3 had not been performed in a vaccination cycle or respective data were not available for this visit, the baseline visit (V0) of the following cycle was used as V3 in case the visit date was within the defined time window. Subgroup analyses were performed based on the patients' DMT treatment at the time of enrollment. Due to relatively low case numbers for some DMTs, these were grouped into larger subgroups according to their mode of action.

The primary endpoint was the number and proportion of patients with an immune response to their last SARS-CoV-2 vaccination at V2 of a vaccination cycle. Two definitions of immune response were used and considered separately. An immune response was assumed if the anti-S1 IgG serum level increased by more than 100 BAU/ml between V0 and V2. Based on neutralizing activity, presence of an immune response was assumed if the test for anti-SARS-CoV-2 NAb was positive at V2 (threshold for positivity: ≥35% IH). Secondary endpoints included the number and proportion of patients with an immune response to their last SARS-CoV-2 vaccination at V1 (if applicable), V2, or V3 of a vaccination cycle based on positive anti-S1-IgG serum levels (threshold for positivity: ≥35.2 BAU/ml). Other secondary endpoints were the absolute values and changes from baseline in SARS-CoV-2-specific immunoglobulin levels, including anti-NCP-IgG [threshold for positivity: ≥1.1 (no unit)], the number and proportion of patients per vaccination cycle, persistence of immune response, frequencies of vaccines used, and patient-assessed vaccination tolerability. As the onset dates of relapse episodes were not documented in this study, it was assumed that the start of HDC treatment marked the onset of relapse. The threshold for a positive anti-influenza A IgG test was ≥22 RU/ml.

All documented data were analyzed descriptively using a statistical software package (SAS, version 9.4, SAS Institute Inc, Cary, USA). Categorical variables were presented as absolute and relative frequencies (percentages were based on the total number of enrolled patients, unless otherwise indicated), continuous variables as mean, standard deviation (SD), median, and range. No statistical tests carried out. Since this was an exploratory study no formal sample size calculation was done. The planned sample size of 200 patients was based on the consideration of how many patients were eligible and willing to participate in this study.

## 3 Results

### 3.1 Study population, MS treatment, and SARS-CoV-2 immunization status at enrollment

A total of 159 patients, predominantly women (66.7%, *n* = 106), were enrolled in the study and included in the data analysis. Premature study termination was documented in seven patients (4.4%), with four patients (2.5%) withdrawing voluntarily, one patient (0.6%) being lost-to-follow-up, and two patients (1.3%) having other reasons. The average duration of MS varied greatly among patients and 19 patients (11.9%) had experienced at least one MS relapse in the 6 months prior to enrollment. Thyroid disease (8.8%, *n* = 14) and depression (7.5%, *n* = 12) were the most frequent comorbidities. Most patients (95.6%, *n* = 152) had not yet had a SARS-CoV-2 infection at enrollment. The patients' age ranged between 19 and 71 years and the mean BMI was in the normal weight range. Details on demographic data and other baseline characteristics of the patients are presented in Table 1.

**Table 1:** Demographics and baseline data.

**Characteristic**	**Total *N* = 159**
Age (years), mean ± SD [range]	41.6 ± 10.6 [19–71]
BMI (kg/m^2^), mean ± SD [range]	24.6 ± 5.7 [16–58]
**Sex**, ***n*** **(%)**	
Female	106 (66.7)
Male	53 (33.3)
**Smoking status**, ***n*** **(%)**	
Never smoker	95 (59.7)
Smoker	36 (22.6)
Ex-smoker	28 (17.6)
Duration of MS (years), mean ± SD [range]	10.1 ± 8.2 [0.1–43.9]
MS relapse(s) within the last 6 months, *n* (%)	19 (11.9)
Treatment with high-dose steroids for relapse within the last 6 months, *n* (%)	16 (84.2)
**Comorbidities**, ***n*** **(%)**	
No comorbidity	86 (54.1)
At least one comorbidity	73 (45.9)
SARS-CoV-2 infection prior to study enrollment, *n* (%)	7 (4.4)^a^
**Influenza immunization status**, ***n*** **(%)**	
Vaccinated against influenza	72 (45.3)
Not vaccinated against influenza	74 (46.5)
**DMT treatment at the time of enrollment**, ***n*** **(%)**	
Anti-CD20 antibodies	53 (33.3)
Other monoclonal antibodies (natalizumab, alemtuzumab)	21 (13.2)
Fumarates (DMF)	25 (89.3)
Injectables	17 (10.7)
S1PR modulators	16 (10.1)
Other agents (e.g. TERI, CLAD)	8 (5.0)
No DMT	19 (11.9)

^a^All patients had recovered from the infection before inclusion into the study.

CLAD, cladribine; DMF, dimethyl fumarate; S1PR, sphingosine-1-phosphate receptor; TERI, teriflunomide.

Missing data: BMI, n = 2; influenza immunization status, n = 13.

At the time of inclusion in the study, 140 patients (88.1%) were being treated with a DMT, most frequently with an anti-CD20 agent (33.3%, *n* = 53; Table 1). The frequencies of the individual DMTs at enrollment are summarized in Supplementary Table S1. Most patients (67.9%, *n* = 108) entered the study in the 1st booster cycle, i.e., before the 1st booster dose, and only five patients (3.1%) in the 2nd or 3rd booster cycle (Table 2). Of the 46 patients (28.9%) who were included in the study during the initial vaccination cycle, 10 patients (6.3%) had not yet been vaccinated.

**Table 2:** SARS-CoV-2 immunization status at enrollment.

**Characteristic**	**Total *N* = 159**
**Vaccination cycle in which the patient entered the study**, ***n*** **(%)**	
Initial vaccination cycle	46 (28.9)
Patient not yet vaccinated	10 (6.3)
Patient had received 1st vaccination	9 (5.7)
Patient had received 2nd vaccination	27 (17.0)
1st booster cycle	108 (67.9)
2nd booster cycle	4 (2.5)
3rd booster cycle	1 (0.6)

### 3.2 Frequency of SARS-CoV-2 vaccinations per patient, vaccines used, and timing of vaccination in patients treated with ocrelizumab

At study end, all 159 patients had completed the initial vaccination cycle, in most cases with a double-dose vaccine (96.9%, *n* = 154), and most patients had also received the 1st booster dose (83.0%, *n* = 132). Further booster cycles were less frequently completed. All booster vaccinations were given as single doses (except for one 2nd booster in one patient). The number of patients per completed cycle and the mean intervals between vaccination doses are presented in Table 3. In patients treated with ocrelizumab, vaccine doses were administered on average between approximately 3.0 and 3.7 months after the last ocrelizumab dose (mean time intervals in days are provided in Table 3).

**Table 3:** Number of patients per completed SARS-CoV-2 vaccination cycle, time interval between vaccinations, and time between last ocrelizumab dose and vaccination.

**Characteristic**	**Total *N* = 159**
**Vaccination cycle completed (by cycle and type of vaccination)**,	
***n*** **(%)**	
Initial cycle	159 (100.0)
Single dose	5 (3.1)
Double dose	154 (96.9)
1st booster cycle	132 (83.0)
Single dose	132 (100.0)
2nd booster cycle	42 (26.4)
Single dose	41 (97.6)
Double dose	1 (2.4)
3rd booster cycle	3 (1.9)
Single dose	3 (100.0)
**Time between vaccinations (days), mean** ±**SD**	
1st and 2nd initial doses (*n* = 154)	39.6 ± 16.7
2nd initial and 1st booster dose (*n* = 132)	179.3 ± 36.9
1st and 2nd booster dose (*n* = 35)	245.3 ± 92.5
2nd and 3rd booster dose (*n* = 3)	248.7 ± 51.1
**Time between the last ocrelizumab dose and vaccination (days)**,	
**mean** ±**SD**	
1st initial dose (*n* = 37)	92.1 ± 37.2
2nd initial dose (*n* = 39)	107.4 ± 48.1
1st booster dose (*n* = 35)	108.7 ± 48.4
2nd booster dose (*n* = 13)	112.5 ± 27.4

Percentages for frequencies of single or double dose vaccinations were based on the number of patients who completed the respective cycle.

Comirnaty^®^ (Pfizer/BioNTech Manufacturing GmbH, Mainz Germany) was the most frequently used vaccine (87.5%, 426/487 vaccinations). Spikevax^®^ (Moderna, Cambridge, MA, USA) was used for 46 (9.4%), Vaxzevria^®^ (Oxford/AstraZeneca, Nijmegen, The Netherlands) for 10 (2.1%), Jcovden^®^ (Janssen-Cilag/Johnson&Johnson, Beerse, Belgium) for 3 (0.6%), and Nuvaxovid^®^ (Novavax, Gaithersburg, MD, USA) for two vaccinations (0.4%). Among the patients with at least three vaccinations, the most frequent vaccine combination was three times Comirnaty^®^ (72.0%, *n* = 95/132) followed by Comirnaty^®^ for the first two and Spikevax^®^ for the third vaccination (16.7%, *n* = 22/132). Other combinations were each documented in less than 5% of the patients.

### 3.3 Immune response to SARS-CoV-2-vaccination

**Figure 2:**
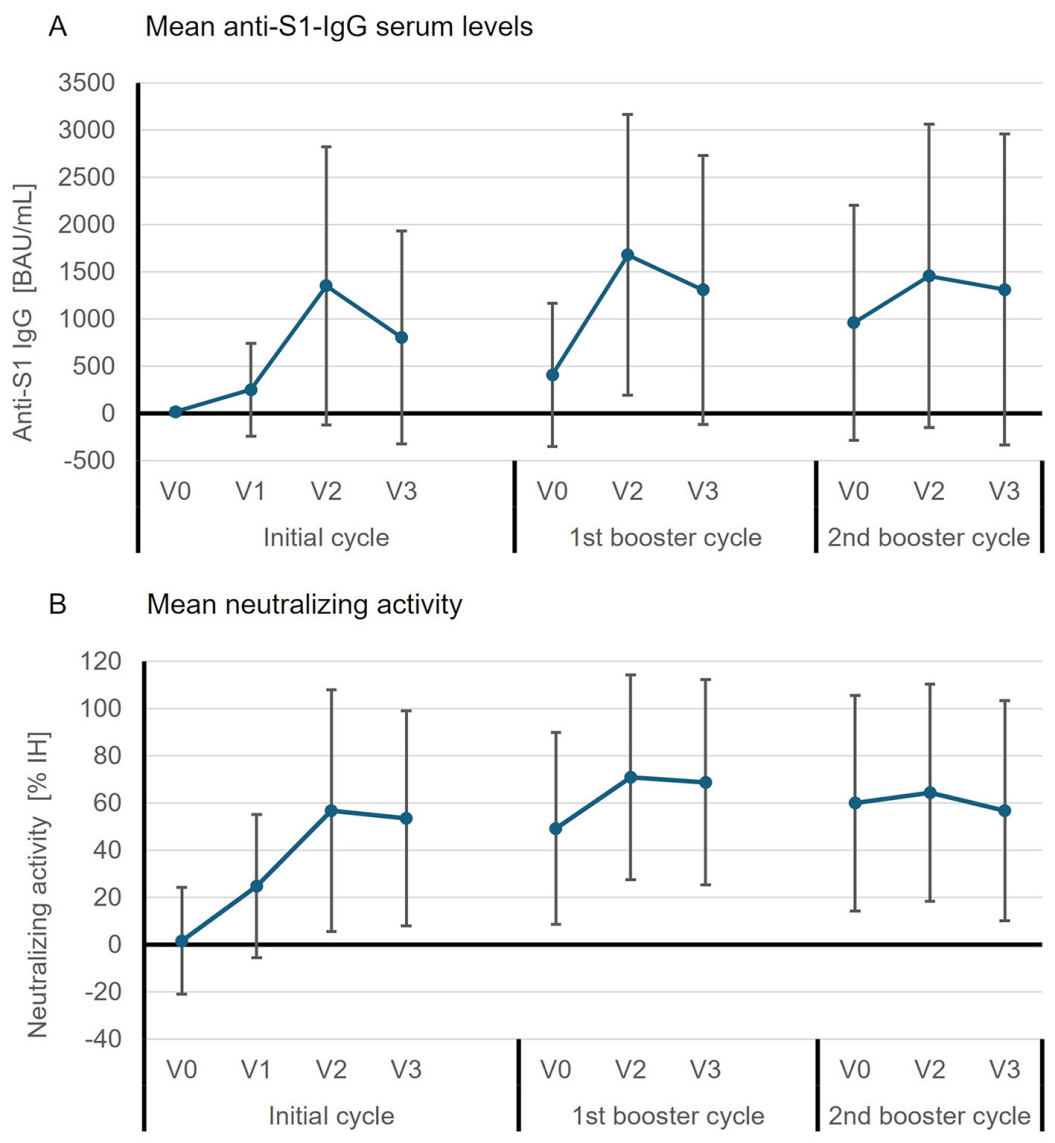
Mean (± SD) absolute values of **(A)** anti-S1-IgG serum levels (BAU/ml) and **(B)** neutralizing activity (% IH, percent inhibition) of anti-S1-IgG per visit and vaccination cycle. Data were available from the following numbers of patients: for the initial cycle *n* = 9 (V0), *n* = 16 (V1), *n* = 40 (V2), *n* = 24 (V3); for the 1st booster cycle *n* = 124 (V0), *n* = 122 (V2), *n* = 106 (V3); for the 2nd booster cycle *n* = 36 (V0), *n* = 28 (V2), *n* = 29 (V3). Neutralizing activity could not be determined in the initial cycle for 1 patient at V0, 1 patient at V1, and 2 patients at V2.

Mean anti-S1-IgG serum levels were well above the cut-off for positivity at all time points after the 1st vaccination of the initial cycle (Figure 2A), with the highest value at V2 of the 1st booster cycle (1,680.6 ± 1,485.8 BAU/ml, *n* = 122). The rate of seropositive patients was 73.8% (*n* = 90/122) at this visit. The proportion of anti-S1-IgG-positive patients was already high at baseline of the 1st booster cycle (67.7%, *n* = 84/124) and before the 2nd booster dose (69.4%, *n* = 25/36). Similarly, neutralizing activity was detectable at all time points after the 2nd initial vaccination, with the highest mean rate of inhibition at V2 of the 1st booster cycle (70.9 ± 43.4% IH) and high inhibition rates still measurable before the 1st and 2nd booster doses (Figure 2B). Overall, 39 patients (24.5%) treated with either ocrelizumab (anti-CD20 antibody) or fingolimod (S1PR modulator) remained seronegative for anti-S1-IgG antibodies at all sample times.

**Figure 3:**
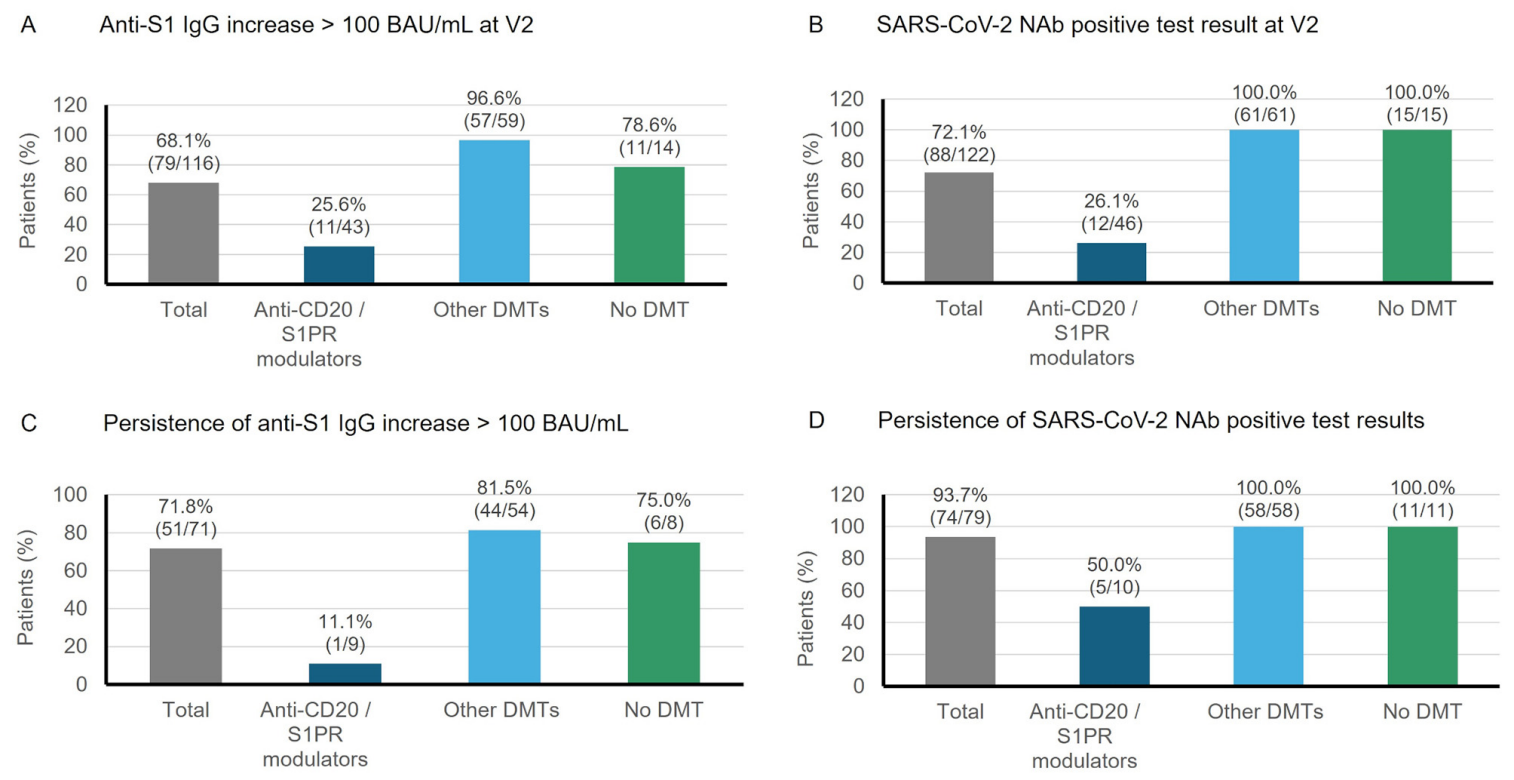
Percentage of patients with immune response at V2 after the 1st booster vaccination based on **(A)** anti-S1-IgG increase >100 BAU/ml from baseline, or **(B)** a positive neutralizing antibody (NAb) test result. Persistence was assumed if **(C)** anti-S1-IgG increase from baseline was >100 BAU/ml or **(D)** NAb test results were positive (≥35% IH) over approximately 3 months or longer (between V2 and V3 of the same cycle). Percentages of patients with persisting immune response were based on the number of patients with an immune response at V2 and anti-S1-IgG or NAb test results available at V3 of the respective cycle.

A sufficiently high number of patients with available anti-S1-IgG values before and after vaccination could only be documented for the 1st booster cycle. Therefore, with regard to the primary endpoint only the results for this cycle are reported here (for frequencies of responders in all cycles see Supplementary Table S2). After the 1st booster (at V2), 68.1% of the patients (*n* = 79/116) were responders based on anti-S1-IgG increase; this response was less frequent in patients treated with anti-CD20 antibodies or S1PR modulators (25.6%, *n* = 11/43) compared to patients treated with other DMTs (96.6%, *n* = 57/59) or patients not receiving DMT (78.6%, *n* = 11/14; Figure 3A). Changes from baseline in anti-S1-IgG were also smallest in the anti-CD20/S1PR subgroup at both V2 and V3 (Table 4). It was observed that five out of seven patients who received an anti-CD20 agent and with a positive response had only recently switched to this DMT (between approximately 3.5 and 7.4 months ago; individual data not shown). Based on NAb seropositivity, the overall response rate was 72.1% (*n* = 88/122) at V2 after the 1st booster dose with 100% responders in the other DMT and no DMT subgroups (Figure 3B). Impaired immune response in patients treated with anti-CD20/S1PR modulators was also reflected in the changes from baseline at V2 and V3 of the 1st booster cycle (Table 4).

**Table 4:** Changes from baseline in anti-S1 IgG antibodies and neutralizing activity in the 1st booster cycle.

	**Anti-S1-IgG (BAU/ml)**				**Neutralizing activity (%IH)**			
	**V2**		**V3**		**V2**		**V3**	
**DMT treatment**	***N***	**Mean** ±**SD**	***N***	**Mean** ±**SD**	***N***	**Mean** ±**SD**	***N***	**Mean** ±**SD**
Total	116	1,327.8 ± 1,321.3	103	902.4 ± 1,369.5	116	20.7 ± 25.4	103	15.5 ± 25.0
Anti-CD20/S1PR	43	93.3 ± 187.1	36	38.2 ± 375.8	43	13.9 ± 25.2	36	5.3 ± 20.9
Other DMTs^a^	59	2,139.8 ± 1,119.4	56	1,489.5 ± 1,524.1	59	24.5 ± 24.2	56	22.5 ± 24.7
No DMT	14	1,697.5 ± 1,248.9	11	742.1 ± 1,101.3	14	25.4 ± 27.7	11	13.6 ± 28.8

^a^Other DMTs: injectables, natalizumab, alemtuzumab, cladribine, dimethyl fumarate, teriflunomide.

Anti-CD20/S1PR, anti-CD20 antibodies and sphingosine-1-phosphate receptor modulators; BAU, binding antibody units; %IH, percent inhibition.

Persisting immune response between V2 and V3 after the 1st booster (median interval: 139 days, range: 97–238 days, *n* = 103) was seen in 71.8% of the responders (*n* = 51/71) based on anti-S1-IgG increase (Figure 3C) and in 93.7% of the responders (*n* = 74/79) based on neutralizing activity (Figure 3D). Persistence was impaired in patients treated with anti-CD20 agents or S1PR modulators (Figures 3C, D). The persistence rate regarding NAb seropositivity was 100% in the no DTM and other DMT subgroups as well as in the three responders treated with S1PR modulators in the anti-CD20/S1PR subgroup, but only 28.6% (*n* = 2/7) among responders treated with anti-CD20 antibodies.

### 3.4 Patient-assessed vaccination tolerability, and treatment of vaccination side effects

**Figure 4:**
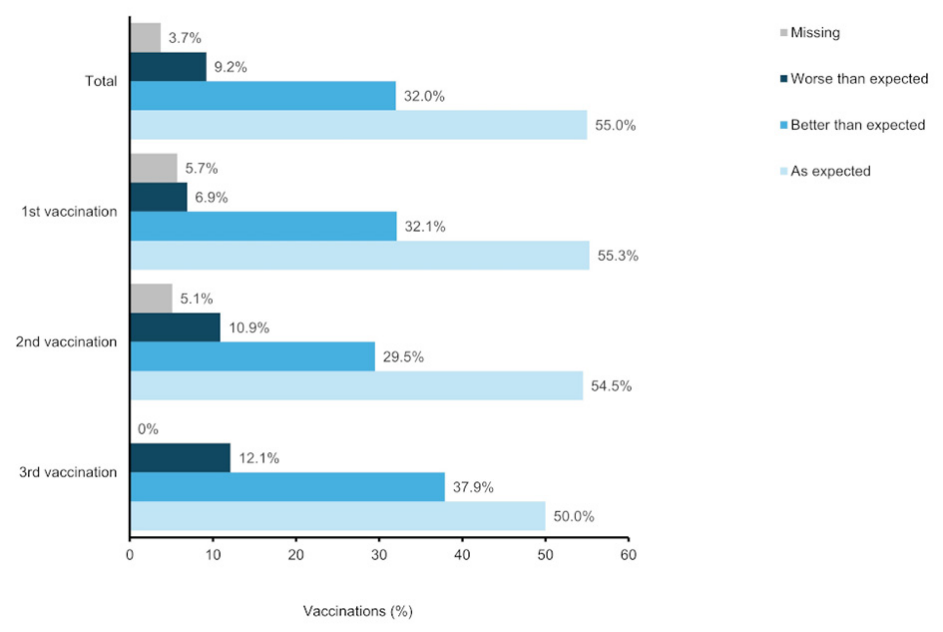
Patient-assessed vaccination tolerability. Percentages were based on the number of vaccinations: 487 vaccination (total), 159 vaccinations (1st vaccination), 156 vaccinations (2nd vaccination), 132 vaccinations (3rd vaccination).

As shown in Figure 4, the tolerability of the 1st vaccination was rated slightly better by the patients than that of the 2nd vaccination (2nd initial dose in most cases) and 3rd vaccination (1st booster dose in most cases).

Drug treatment, usually with non-steroidal anti-inflammatory drugs, to prevent or treat possible vaccination side effects was rarely necessary (9.0%, 44/487 vaccinations) and more common post-dose (97.7%, 43/44 vaccinations) than pre-dose (13.6%, 6/44 vaccinations).

### 3.5 SARS-CoV-2 infection

Five patients (3.1%) had been infected with the SARS-CoV-2 virus prior to any SARS-CoV-2 vaccination and 58 patients (36.5%) had at least one documented SARS-CoV-2 infection after vaccination. The percentage of patients with breakthrough infection was similar between patients treated with anti-CD20/S1PR modulators (37.7%, *n* = 26/69) and other DMTs (36.6%, *n* = 26/71), and slightly lower in the no DMT subgroup (31.6%, *n* = 6/19; Supplementary Figure S1). The hospitalization rate was low (1.6%, 1/62 infections). The median time to diagnosis of SARS-CoV-2 infection after any SARS-CoV-2 vaccination was 108.5 days (range: 7–282 days). An additional 11 patients (6.9%) without documented SARS-CoV-2 infection most likely had a silent infection as they had detectable anti-NCP antibodies during the study period.

Most infections were reported during the omicron waves in Germany from January to April 2022 and in June/July 2022. All patients with breakthrough infections were fully vaccinated and 51 patients (87.9%) had also received at least one booster vaccination. The frequency of positive anti-NCP-IgG tests peaked at V3 of the 1st booster cycle, which correlated with the summer omicron wave in Germany in 2022.

### 3.6 MS relapse and EDSS

Seven patients (4.4%) experienced an MS relapse during the study period and in the follow-up phase after a SARS-CoV-2 vaccination. Of these, 6 patients received HDC treatment for relapse. Two of the patients had also received HDC for MS relapse at some time after a SARS-CoV-2 vaccination prior to study participation. Overall, 11 patients (6.9%) received HDC at least once after a SARS-CoV-2 vaccination (before or after enrollment into the study), mainly after the 2nd dose (*n* = 10), two patients after the 3rd dose, and one patient after the 5th dose. The median time to start HDC treatment after any SARS-CoV-2 vaccination was 140 days (range: 19–199 days). Three patients with at least one MS relapse prior to enrollment did not receive HDC, thus the temporal relationship with a SARS-CoV-2 vaccination was unknown. Another nine patients with at least one MS relapse in the last 6 months prior to enrollment received HDC treatment, but the treatment started prior to any SARS-CoV-2 vaccination.

There was no evidence of a change in disability status following SARS-CoV-2 vaccination as measured by the EDSS. The median EDSS was 2.0 at most or all visits of all three cycles (initial, 1st and 2nd booster cycles; Supplementary Table S3), but EDSS data were missing for up to 55.5% of patients per visit.

### 3.7 General immunization status and anti-influenza A virus antibody titers

Vaccination certificates were available for 146 patients (91.8%). Most of these patients had received vaccinations against diphtheria (95.9%, *n* = 140/146), tetanus (95.9%, *n* = 140/146), poliomyelitis (90.4%, *n* = 132/146), and pertussis (79.5%, *n* = 116/146). Other frequent vaccinations (i.e., in >50% of the patients) were those against measles, hepatitis B, rubella, mumps, and tick-borne encephalitis. Vaccination coverage for influenza and hepatitis A was < 50%; other vaccinations were even less frequent (see Supplementary Table S4 for frequencies of all documented vaccinations).

Except in one patient, anti-influenza A virus IgG antibodies were detectable in all patients: 151 patients (95.0%) had at least one positive antibody test and seven patients (4.4%) had at least a borderline test result at some point during the study. Frequencies of anti-influenza A virus IgG test results are provided per cycle and visit in Supplementary Table S5.

## 4 Discussion

People with chronic autoimmune diseases such as MS were faced with particular challenges at the outbreak of the COVID-19 pandemic in 2020. Initially, the risk factors for infection or disease severity in this patient group were unknown. This applied also to the immune system's response to the virus and the soon-to-be available vaccines, especially in the context of immunomodulatory and immunosuppressive therapies. In this epidemiological study, we therefore collected data in routine clinical practice on SARS-CoV-2 vaccination, immunization status, humoral immune response, and infection rate in MS patients over several vaccination cycles.

Immune response to the SARS-CoV-2 vaccination was determined by an increase in anti-S1-IgG antibodies of at least 100 BAU/ml between baseline and V2 of a cycle as well as – separately—by the presence of a positive NAb test at V2 of a cycle. All patients with an immune response based on anti-S1-IgG were NAb positive at V2 of the respective cycle. However, the proportions of responders based on anti-S1-IgG increase were lower than the proportions of responders based on NAb positivity in the 1st and 2nd booster cycles. This can be explained by the high antibody titers that persisted from the previous vaccination in a high proportion of patients at the baseline visits of the booster cycles. In patients with an already high antibody titer, a booster dose may not have provoked an increase of >100 BAU/ml and this threshold for the assessment of immune response may therefore not have been ideal. An increase of >100 BAU/ml had originally been chosen because the value of 100 BAU/ml was well above the laboratory threshold for seropositivity. Several studies in healthcare workers or other general populations have identified minimum levels of protective SARS-CoV-2 antibody titers based on spike protein-specific or total antibody concentrations (14–16). However, no standardized threshold at which protection against SARS-CoV-2 has been established to date, especially not for patients with MS.

Analysis of the data from a randomized trial showed that a vaccination efficacy against symptomatic infection of 80% was achieved 28 days after the second vaccine dose with an anti-spike IgG level of 264 BAU/ml (15). Mean anti-S1-IgG levels in the present study were well above this threshold at V2 during each cycle (≥1,680.6 BAU/ml). In a retrospective study on immune response in cladribine-treated patients with MS, 94% of the patients reached seropositivity with mean titers of 702 ± 906 BAU/ml in patients vaccinated ≤ 18 weeks and 1,207 ± 791 BAU/ml in patients vaccinated >18 weeks after the last cladribine dose; humoral response was deemed adequate in these patients (17). In comparison, a study in 1,750 healthcare workers showed anti-SARS-CoV-2-spike IgG titers of up to 1,144.4 BAU/ml after threefold vaccination and 33 days (geometric mean) after the last immunization (18). Our anti-S1-IgG levels were also in the same range as those found in 39 MS patients and 273 control subjects (healthcare workers) in an exploratory case-control study (19).

We observed impaired immune response based on anti-S1-IgG increase as well as neutralizing activity in patients treated with anti-CD20 antibodies and S1PR modulators. In this subgroup, the persistence of humoral immune response was also much lower. Several recent studies have shown impaired immune response to SARS-CoV-2 vaccination in patients treated with these types of DMTs for MS or other medical conditions (20–24). Despite low antibody titers against the spike protein, patients treated with anti-CD20 antibodies may still achieve some degree of protection through T-cell response (25, 26). Suspension of these therapies may improve the humoral response and a gap of 6 months between the last anti-CD20 medication and receiving a vaccination has been discussed (27). However, a meta-analysis revealed that rates of seroconversion may even be reduced more than 12 months after the last anti-CD20 antibody treatment (28). Intervals between the last ocrelizumab dose before a SARS-CoV-2 vaccination determined in the current study were on average much shorter than 6 months. Patients treated with other DMTs or DMT-untreated patients seemed to produce adequate antibody titers after SARS-CoV-2 vaccination. Similar observations were also reported in other studies (29, 30).

Anti-CD20 therapy has also been associated with impaired immune response to other types of vaccinations (28). For example, reduced humoral immune response to the influenza A vaccination has been reported in ofatumumab-treated patients with relapsing MS (31) or in rituximab-treated patients with hematological malignancies (32). Interestingly, we observed that all but one patient had positive or at least borderline positive tests for anti-influenza A IgG at almost all sample times, including the 39 patients treated with anti-CD20 antibodies or S1PR modulators, who otherwise remained seronegative for anti-S1-IgG antibodies during the entire study period. Whether this observation indicates a better immune response to the influenza A than the SARS-CoV-2 vaccination in these patients remains unclear due to our limited data regarding influenza A vaccinations (e.g., vaccination dates were not collected). In addition, not all patients were vaccinated against influenza (according to the vaccination certificates) and positive titers could also result from previous influenza infections.

Comirnaty^®^ was the most frequently used vaccine among the study patients, most likely because this vaccine was the most frequently available or recommended vaccine in Germany in 2021. The mean time intervals between vaccinations approximated the vaccination intervals recommended for this vaccine by the German Standing Committee on Vaccinations (STIKO) in 2021 (33). It should be noted that the recommendations by the STIKO were continuously updated during the study period (34).

Since our study included only patients who either had already received one or more SARS-CoV-2 vaccinations or wanted to be vaccinated against this virus, a bias in the study population was to be expected. For example, older patients or patients with a higher disability status might have been more inclined to be vaccinated than younger or healthier patients. Such a bias was not observed. The average age of the study population was close to that of the German population in 2021 (41.6 vs. 44.7 years) (35), and the patients had, on average, a low level of disability. The gender distribution in this study reflects the higher prevalence of this disease in women in general (36).

In contrast to other studies (37, 38), we did not observe a higher breakthrough infection rate in patients treated with anti-CD20 antibodies or S1PR modulators than in those treated with other DMTs. A possible reason for this observation may be the protective effect of a compensatory T-cell immune response (25, 26). The absolute rate of COVID-19 infection after vaccination was higher compared to that found in an observational prospective cohort study in vaccinated German healthcare workers (36.5 vs. 9.7%) (39), but similar to the omicron breakthrough infection rate of 36% found in a cohort of patients with MS (38). High rates of >40% asymptomatic SARS-CoV-2 infections have been found in systematic literature reviews and meta-analyses in global populations (40, 41). A systematic review in patients with MS (42) found a considerably lower rate of 5.3% asymptomatic cases, which roughly corresponds to the proportion of anti-NCP-IgG seropositive patients (6.9%) without reported COVID-19 infections in our study.

The rate of HDC-treated MS relapses in patients after any SARS-CoV-2 vaccination (before or after enrollment in the study) was lower than the post-vaccination relapse rate found in another observational study with a distinctly higher sample size of 2,466 patients (6.9 vs. 13.8%) (43). However, the total relapse rate after at least one SARS-CoV-2 vaccination may have been slightly higher, as the temporal connection to a vaccination could not be determined in three patients who were not treated with HDC for MS relapse before enrollment. In addition, there was one patient without HDC treatment but with a relapse after enrollment and after vaccination. Most relapse episodes occurred after the 2nd dose. In the study by Fneish et al. (43) also most patients had relapses after the 2nd or 3rd dose, but the median time interval between vaccination and relapse was shorter than in our study (8 weeks compared to approximately 20 weeks).

Limiting factors of this study include the fact that patients could enter the study before or after any SARS-CoV-2 vaccination. At the start of the study in July 2021, the German SARS-CoV-2 vaccination program had already been running for 6 months. Many MS patients who were eligible for the study entered the study in the 1st booster cycle after having already received the first complete SARS-CoV-vaccination. Therefore, not enough data could be collected for the initial vaccination cycle to conduct a robust longitudinal observation over several cycles with the same sample of patients. Furthermore, the planned sample size of 200 patients was not achieved, which may be attributable to a general decline in the willingness to vaccinate against SARS-CoV-2 with the appearance of the omicron variant and the changing recommendations of the STIKO regarding the frequency and target groups of booster vaccinations (44). As the planned sample size was not based on a formal sample size calculation, the achieved sample size of 159 patients was deemed sufficient to draw meaningful conclusions with regard to the objectives of the study. Comparisons between the treatment subgroups were made purely on a descriptive basis and may be limited by the relatively small subgroup of DMT-untreated patients compared to the other two subgroups. Patient-reported outcomes such as the patient-assessed vaccine tolerability are prone to selection or language bias.

In conclusion, the results of this epidemiological cohort study showed that patients with MS, who were either treated with a DMT or not, were able to produce high titers of spike-protein-related antibodies and sufficient neutralizing activity after SARS-CoV-2 vaccinations. Furthermore, persistence of antibody levels and neutralizing activity was seen over a period of at least 3 months in a high proportion of responders. Treatment with anti-CD20 antibodies and S1PR modulators may lead to impaired humoral immune response and reduced neutralizing activity. In clinical practice, monitoring SARS-CoV-2 antibody levels in patients taking these DMTs and careful timing of certain DMTs prior to vaccination may increase the chances for successful vaccination against SARS-CoV-2 in these patients.

## Data Availability

The raw data supporting the conclusions of this article will be made available by the authors, without undue reservation.
