# Supplementary material for: A multicenter, prospective cohort study on the anti-SARS-CoV-2 vaccination response in patients with multiple sclerosis in Germany
Source: Front Neurol. 2025 Oct 29;16:1674742. doi: 10.3389/fneur.2025.1674742 (PMC12606583; doi:10.3389/fneur.2025.1674742)

Supplementary Material

# Supplementary Tables

**Supplementary Table 1.** Frequency of individual DMTs at enrollment

| DMT, n (%) | Total N = 159 |
| --- | --- |
| Anti-CD20 antibodies: |  |
| Ocrelizumab | 43 (81.1) |
| Rituximab | 7 (13.2) |
| Ofatumumab | 3 (5.7) |
| Other monoclonal antibodies: |  |
| Natalizumab | 18 (100.0) |
| Alemtuzumab | 3 (37.5) |
| Fumarates: |  |
| Dimethyl fumarate | 25 (89.3) |
| Injectables: |  |
| Glatiramer acetate, once daily | 2 (11.8) |
| Glatiramer acetate, 3 times per week | 6 (35.3) |
| Peginterferon beta-1a, IM | 1 (5.9) |
| Interferon beta-1a, IM | 3 (17.6) |
| Interferon beta-1a, SC | 3 (17.6) |
| Interferon beta-1b, SC | 2 (11.8) |
| S1PR modulators: |  |
| Fingolimod | 14 (87.5) |
| Siponimod | 2 (12.5) |
| Other agents: |  |
| Cladribine | 5 (62.5) |
| Teriflunomide | 3 (10.7) |

DMT, disease-modifying therapy; IM, intramuscular; n/N, number of patients;
SC, subcutaneous; S1PR, sphingosine-1-phosphate receptor.

**Supplementary Table 2.** Anti-S1-IgG [BAU/mL] serum levels and changes from baseline per vaccination cycle

|  |  |  | **Absolute value** | | **Change from V0** | | | |
| --- | --- | --- | --- | --- | --- | --- | --- | --- |
|  | **N** | **Nmiss** | **Mean ± SD** | |  | **N** | **Nmiss** | **Mean ± SD** |
| **Initial cycle** | | |  | |  |  |  |  |
| V0 | 9 | 0 | 18.7 ± 35.4 | |  |  |  |  |
| V1 | 16 | 0 | 251.9 ± 492.0 | |  |  |  |  |
| V2 | 40 | 0 | 1353.0 ± 1472.8 | |  | 9 | 31 | 1545.6 ± 1409.5 |
| V3 | 24 | 0 | 804.3 ± 1128.4 | |  | 3 | 21 | 1074.6 ± 947.5 |
| **1st booster cycle** | | | |  |  |  |  |  |
| V0 | 124 | 1 | 410.1 ± 758.5 | |  |  |  |  |
| V2 | 122 | 0 | 1680.6 ± 1485.8 | |  | 116 | 6 | 1327.8 ± 1321.3 |
| V3 | 106 | 1 | 1309.7 ± 1423.8 | |  | 103 | 4 | 902.4 ± 1369.5 |
| **2nd booster cycle** | | | |  |  |  |  |  |
| V0 | 36 | 0 | 960.7 ± 1243.7 | |  |  |  |  |
| V2 | 28 | 1 | 1457.8 ± 1604.9 | |  | 26 | 3 | 592.1 ± 964.2 |
| V3 | 29 | 0 | 1313.1 ± 1646.3 | |  | 27 | 2 | 492.6 ± 1342.7 |

Titers >3840 BAU/mL were included in the analysis with a value of 3840 BAU/mL (63 timepoints in 34 patients). Titers <3.2 BAU/mL were included in the analysis with a value of 3.2 BAU/mL (81 timepoints in 32 patients).
Anti-S1-IgG, anti-SARS-CoV-2 spike protein (S1 domain) antibody (immunoglobulin G); N, number of patients; Nmiss, number of patients with missing data.

**Supplementary Table 3.** Expanded Disability Status Scale (EDSS) score per vaccination cycle and visit

|  | **Absolute values** | | | |
| --- | --- | --- | --- | --- |
|  | **N** | **Nmiss** | **Mean ± SD** | **Median (range)** |
| **Initial cycle (I)** |  |  |  |  |
| V0 | 10 | 0 | 2.0 ± 1.1 | 2.3 (0.0-3.0) |
| V1 | 13 | 3 | 1.5 ± 0.8 | 2.0 (0.0-2.5) |
| V2 | 31 | 9 | 2.4 ± 1.8 | 2.0 (0.0-6.5) |
| V3 | 13 | 11 | 1.7 ± 0.9 | 1.5 (0.0-3.5) |
| **1st booster cycle** |  |  |  |  |
| V0 | 117 | 8 | 2.4 ± 2.1 | 2.0 (0.0-10.0) |
| V2 | 73 | 50 | 2.0 ± 1.9 | 2.0 (0.0-8.0) |
| V3 | 63 | 46 | 2.2 ± 1.9 | 2.0 (0.0-7.5) |
| **2nd booster cycle** |  |  |  |  |
| V0 | 23 | 13 | 2.5 ± 1.9 | 2.0 (0.0-6.0) |
| V2 | 13 | 16 | 3.2 ± 1.9 | 2.5 (1.0-6.5) |
| V3 | 17 | 14 | 2.4 ± 1.7 | 2.0 (0.0-6.0) |

**Supplementary Table 4.** Frequencies of vaccinations against different pathogens other than SARS-CoV-2 documented from patients’ vaccination certificates

| Vaccination, n (%) | Total N = 159 |
| --- | --- |
| Patients with vaccination certificate available | **146** |
| Diphtheria | 140 (95.9) |
| Tetanus | 140 (95.9) |
| Poliomyelitis | 132 (90.4) |
| Pertussis | 116 (79.5) |
| Measles | 92 (63.0) |
| Hepatitis B | 89 (61.0) |
| German measles | 85 (58.2) |
| Mumps | 85 (58.2) |
| FSME | 84 (57.5) |
| Influenza | 72 (49.3) |
| Hepatitis A | 66 (45.2) |
| Pneumococcal | 43 (29.5) |
| Meningococcal | 21 (14.4) |
| Varicella | 21 (14.4) |
| Haemophilus influenzae type B | 19 (13.0) |
| Human papillomavirus | 13 (8.9) |
| Rotavirus | 6 (4.1) |

Percentages are based on the number of patients for whom vaccination certificates were available.

**Supplementary Table 5.** Frequencies of anti-influenza A virus IgG test results per vaccination cycle and visit

|  | **Test result** | | | |
| --- | --- | --- | --- | --- |
|  | **N** | **Positive** | **Borderline** | **Negative** |
| **Initial cycle (I)** |  |  |  |  |
| V0 | 9 | 8 (88.9) | 1 (11.1) | 0 (0.0) |
| V2 | 40 | 35 (87.5) | 4 (10.0) | 1 (2.5) |
| V3 | 24 | 21 (87.5) | 2 (8.3) | 1 (4.2) |
| **1st booster cycle** |  |  |  |  |
| V0 | 124 | 118 (95.2) | 3 (2.4) | 3 (2.4) |
| V2 | 122 | 114 (93.4) | 8 (6.6) | 0 (0.0) |
| V3 | 106 | 101 (95.3) | 3 (2.8) | 2 (1.9) |
| **2nd booster cycle** |  |  |  |  |
| V0 | 36 | 32 (88.9) | 2 (5.6) | 2 (5.6) |
| V2 | 28 | 25 (89.3) | 3 (10.7) | 0 (0.0) |
| V3 | 29 | 25 (86.2) | 2 (6.9) | 2 (6.9) |
| **3rd booster cycle** |  |  |  |  |
| V0 | 2 | 2 (100.0) | 0 (0.0) | 0 (0.0) |
| V2 | 2 | 2 (100.0) | 0 (0.0) | 0 (0.0) |
| V3 | 2 | 2 (100.0) | 0 (0.0) | 0 (0.0) |

Percentages are based on the number of patients with anti-influenza A virus IgG test evaluation available at the respective visit of the respective cycle.
^a^ Test results anti-influenza A IgG [RU/mL]: ≥22 (positive); ≥16 - <22 (borderline); <16 (negative).

# Supplementary Figures

**Supplementary Figure 1.** Frequency of SARS-CoV-2 breakthrough infections


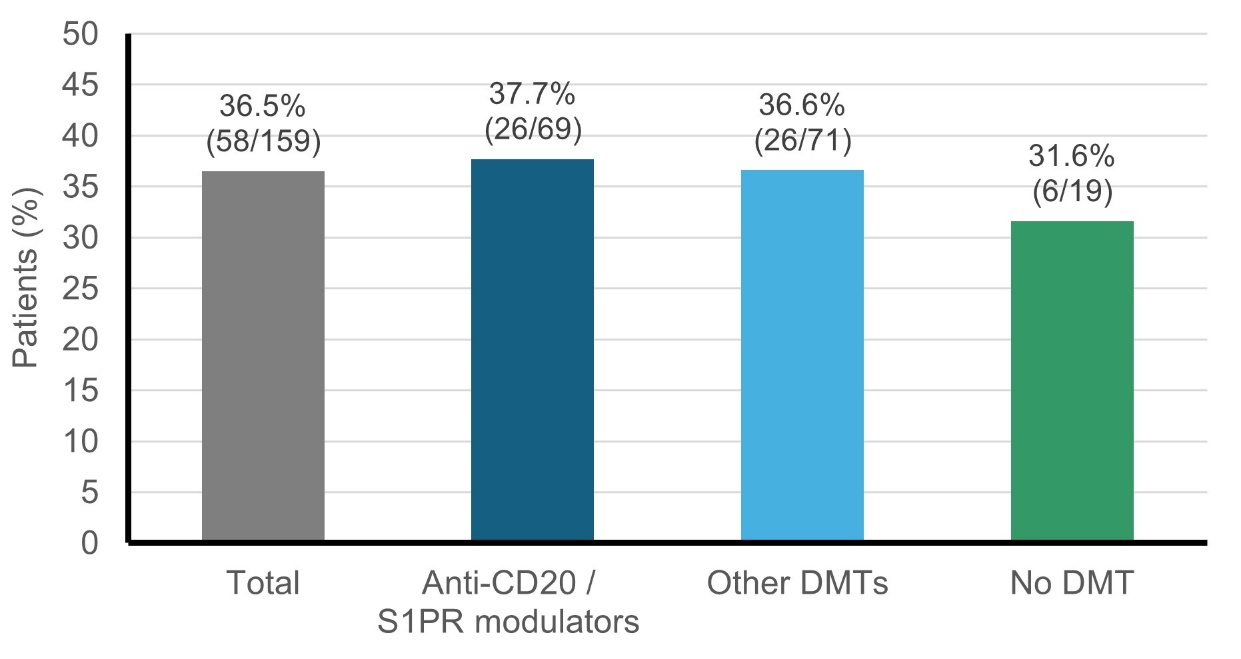

Supplement: Supplementary file 1 [file Table_1.docx]
